# Geographical variation in malignant and benign/borderline brain and CNS tumor incidence: a comparison between a high-income and a middle-income country

**DOI:** 10.1007/s11060-020-03595-5

**Published:** 2020-08-19

**Authors:** Miriam Wanner, Sabine Rohrmann, Dimitri Korol, Nino Shenglia, Teimuraz Gigineishvili, David Gigineishvili

**Affiliations:** 1 Cancer Registry Zurich, Zug, Schaffhausen and Schwyz, University Hospital Zurich, Zurich, Switzerland; 2 Division of Chronic Disease Epidemiology, Epidemiology, Biostatistics and Prevention Institute, University of Zurich, Zurich, Switzerland; 3 Department of Neurology & Neurosurgery, Tbilisi State University, Tbilisi, Georgia

**Keywords:** Brain tumors, CNS tumors, Epidemiology, Benign/borderline, Malignant

## Abstract

**Purpose:**

There is large variability in reported incidence rates of primary brain/CNS tumors across the world, with mostly higher rates in higher-income countries. The aim was to compare malignant and benign brain/CNS tumor incidence between Zurich (Switzerland), a high-income country, and Georgia, a lower middle-income country.

**Methods:**

For the period March 2009 to February 2012, we extracted the following tumors based on topography according to ICD-O3: C70.0–C72.9, and C75.1 (pituitary gland). Data were categorized into histology groups based on the WHO 2007 histological classification. Age-standardized rates per 100,000 person-years were calculated by subgroups.

**Results:**

We included 1104 and 1476 cases of primary brain/CNS tumors for Zurich and Georgia, respectively. Mean age of patients was significantly lower in Georgia compared to Zurich (50.0 versus 58.3 years). Overall age-standardized incidence rates for malignant and benign brain/CNS tumors were 10.5 (95% CI 9.9–11.0) for Georgia and 23.3 (95% CI 21.9–24.7) for Zurich with a ratio of benign to malignant tumors of 1.656 for Georgia and 1.946 for Zurich. The most frequent histology types were meningiomas in both regions, followed by glioblastomas in Zurich, but pituitary tumors in Georgia.

**Conclusion:**

Age-adjusted incidence rates of brain/CNS tumors were considerably higher in Zurich compared to Georgia, both for benign and malignant tumors, which is in line with other studies reporting higher rates in high-income than in low- and middle-income countries. The frequency distribution may be related to differences in diagnosing techniques and the population age structure.

**Electronic supplementary material:**

The online version of this article (10.1007/s11060-020-03595-5) contains supplementary material, which is available to authorized users.

## Introduction

Primary brain and central nervous system (brain/CNS) tumors include a collection of neoplasms (gliomas, meningiomas, neuromas etc.) that vary widely by the degree of malignancy. Aside from increasing age, high-dose radiation, and some hereditary syndromes, no risk factors for these tumors have been established and their etiology remains largely unknown [1]. They are rather infrequent with respect to incidence rates, but mortality is high. Since brain/CNS tumors frequently occur at a younger age than other tumors, they have a strong impact on years of potential life lost due to cancer. In Switzerland, for the years 2003–2007, brain/CNS tumors ranked 15 with respect to incidence, but second with respect to years of potential life lost in men [2].

Data from 39 countries indicate large variability in the incidence rates of brain/CNS cancer with a fivefold difference between the highest (mainly in Europe) and the lowest rates (mainly in Asia) [3]. The observed variation between countries might be partly due to differences in health systems infrastructure, access to care, and the availability of diagnostic services. However, quality and availability of incidence data in low- and middle-income countries, e.g. due to differences in registration/reporting, need critical consideration as well.

Primary benign and borderline brain/CNS tumors rarely invade adjacent tissue and do not metastasize to other parts of the body as the more aggressive malignant brain/CNS tumors do [4]. This is one of the reasons why most cancer registries only collect information on malignant brain/CNS tumors. Still, benign brain/CNS tumors can cause serious health problems, mainly because of the anatomical location, which can cause damage by growing into and putting pressure on other parts of the brain, and therefore can be as deadly as malignant tumors. As most cancer registries do not collect information on benign tumors, Bell et al. estimated the incidence of benign brain tumors by using the US ratio of benign to malignant brain tumors of 2.114 [5]. However, they also stated that this rate might differ geographically due to genetic and environmental factors.

The main aim of our study was to compare brain/CNS tumor incidence between a cancer registry in Switzerland, a high-income country with a universal health care system that provides basic health care for all, and Georgia, a former Soviet republic that became independent in 1991 and is considered a lower middle-income country by the World Bank (https://data.worldbank.org/?locations=GE-XN). Since information on differences between countries of different economic development are rare, we put a specific focus on differences in the incidence of benign brain/CNS tumors.

## Methods

### Zurich cancer registry

Zurich is the largest canton in Switzerland with a population of about 1.5 million in 2017 [6]. The Cancer Registry Zurich, Zug, Schaffhausen and Schwyz was established in 1980 in the canton of Zurich. Since 2011, it also registers cancer cases for the canton of Zug and since 2020 for the cantons of Schaffhausen and Schwyz. Each year, about 120 incident malignant brain/CNS tumors and about 200 benign tumors are registered in the canton of Zurich. The registry receives notifications (patient information, tumor characteristics) from pathology and hematology laboratories, hospitals, and physicians as well as death certificates from the Swiss Federal Statistical Office [7]. A completeness of 93.4% for brain/CNS tumors three years after diagnosis was estimated for Zurich [8], which is above the international level for satisfactory completeness of 90%.

### Georgia brain tumor registry

The Georgian Brain Tumor Data Bank was established in 2008 by one author (DG) and team from the Department of Neurology & Neurosurgery at the Tbilisi State University and was supported by Rustaveli National Science Foundation. Georgia had a population of about 3.7 million in 2018 (https://ec.europa.eu/eurostat/documents/4031688/9684146/KS-01‑19‑056-EN-N.pdf/c3f8811c-3793-48aa-befa-b8ad753f1131). Data were collected between March 1, 2009 and February 29, 2012 from fifteen different hospitals, which provide neurosurgical and neuroradiological services and numerous ambulatory-based CT and MRI units in three large cities (Tbilisi, Kutaisi and Batumi). Active case ascertainment captured all cases of newly diagnosed brain/CNS tumors. More information about case ascertainment and procedures has been published previously [9, 10].

### Data extraction and classification

We extracted the following tumors based on topography of primary sites according to ICD-O3: C70.0–C72.9 and C75.1 (pituitary gland). For Georgia, also tumors of topography C75.2 (craniopharyngeal duct, morphology code 9350) were included. These are not systematically registered in Zurich and were therefore excluded from the Zurich dataset. However, we wanted to be as complete as possible for Georgia and therefore included them in the Georgian data.

We compared incidence of brain/CNS tumors for the period March 1, 2009 to February 29, 2012. The dataset includes information on sex, date of diagnosis, age at diagnosis, ICD-10 code, topography, morphology, and behavior.

Data were categorized into histology groups based on the WHO 2007 histological classification [11], which has also been used in the analyses of the Central Brain Tumor Registry of the United States (CBTRUS) [12]. Furthermore, gliomas were defined as a group of neuroepithelial tissue tumors including all types of astrocytomas, glioblastomas, oligodendrogliomas, oligoastrocytomas, and ependymomas according to CBTRUS [12].

### Statistical analysis

Age standardization was performed based on five-year age groups across the whole age spectrum (18 groups from 0–4 to 85 + years) and standardized to the US 2000 population [13] in order to provide direct comparability of our incidence rates to those of CBTRUS [12]. CBTRUS represents one of the largest datasets of primary brain tumors in the world. In Online Resource 1, incidence rates were further adjusted to the World standard population [14], the WHO standard population [14], and the European standard population (1976) [15] for comparison with studies using one of these standard populations. Age-standardized rates per 100,000 person-years were calculated. For the denominator, we used the populations of Georgia und Zurich, respectively, summed over the years 2009, 2010 and 2011.

All analyses were performed using STATA 13 (Stata Corporation, College Station, TX; USA). The *t* test for continuous and the χ^2^ test for categorical data were used to compare the patient and tumor characteristics between countries. The command “distrate” was used to calculate age-standardized incidence rates overall and for sub groups of the study population [16]. In addition to age-standardized rates, this command calculates efficient interval estimates using formulas developed by Tiwari et al. [17]. Standardized incidence rate ratios (SRR) and 95% confidence intervals were used to compare rates between sub groups according to the method described by Boyle and Parkin [18].

## Results

### Characteristics

Table 1 displays the patient and tumor characteristics. 1104 and 1476 cases of primary brain/CNS tumors were included in the study for Zurich and Georgia, respectively. Mean age of patients was significantly lower in Georgia compared to Zurich (50.0 versus 58.3 years). In both regions, the proportion of female patients was higher than the proportion of male patients. The proportion of malignant tumors was around one third in both regions, but the percentage of gliomas among all brain tumors was higher in Zurich than in Georgia (31.7% vs 19.3%, Table 1). Stratification by tumor behavior and sex revealed a similar distribution of benign/borderline and malignant tumors in men, whereas in women, the proportion of benign/borderline tumors was more than twice (Georgia) or three times (Zurich) as high as the proportion of malignant tumors.

**Table 1:** Descriptive characteristics of brain/CNS tumor data from Georgia and Zurich (Switzerland), March 1, 2009 to February 29, 2012

	Georgia		Zurich		p-value
	N		N		
Total number of cases	1476		1104		
Mean age (years (SD))	1468	50.0 (17.7)	1104	58.3 (19.5)	*< 0.001*
Sex, N (%)	1391		1104		
Male		588 (42.3%)		470 (42.6%)	0.88
Female		803 (57.7%)		634 (57.4%)	
Tumor behavior, N (%)	608		1104		0.08
Benign/borderline		378 (62.2%)		733 (66.4%)	
Malignant		230 (37.8%)		371 (33.6%)	
Gliomas, N (%)	855		1104		*< 0.001*
Glioma		165 (19.3%)		350 (31.7%)	
No glioma		690 (80.7%)		754 (68.3%)	
Tumor behavior by sex, N (%)	1383		1104		
Male benign/borderline		126 (9.1%)		246 (22.3%)	
Male malignant		119 (8.6%)		224 (20.3%)	
Male unspecified		340 (24.6%)		–	
Female benign/borderline		239 (17.3%)		487 (44.1%)	
Female malignant		111 (8.0%)		147 (13.3%)	
Female unspecified		448 (32.4%)		–	

Significance level was set to p < 0.05

*SD* standard deviation

In Zurich, 4.1% of the brain/CNS tumors were not histologically classified (morphology code 8000); this proportion was 42.3% in Georgia. The proportion of histologically verified cases was 52.0% for benign/borderline and 86.5% for malignant brain/CNS tumors in Zurich (overall 63.6%). In Georgia, overall 37.0% of cases were histologically verified.

### Incidence rates

The age-adjusted incidence rates of primary malignant and non-malignant brain/CNS tumors in Zurich and Georgia were 23.3 and 10.5 per 100,000 person-years, respectively (Table 2). While the incidence rate for men and women was not significantly different in Georgia, women had a significantly higher incidence rate compared to men in Zurich. In both regions, the incidence rate for benign/borderline tumors was significantly higher than the one for malignant tumors, with a ratio of benign/borderline to malignant tumors of 1.946 in Zurich and of 1.656 in Georgia. However, simultaneous stratification by tumor behavior and sex revealed a similar incidence rate irrespective of tumor behavior in men, whereas in women, the incidence rates were more than twice (Georgia) and three times (Zurich) as high for benign/borderline compared to malignant tumors. Online Resource 1 shows the age-adjusted incidence rates using different standard populations to allow for a comparison with published data from other regions.

**Table 2:** Age-adjusted incidence rates of brain/CNS tumors in Georgia and Zurich (Switzerland), March 1, 2009 to February 29, 2012

	Georgia		Zurich	
	AIR	95% CI	AIR	95% CI
Overall	10.5	9.9, 11.0	23.3	21.9, 24.7
Sex				
Men	9.2	8.5, 10.0	21.1	19.2, 23.2
Women	10.5	9.7, 11.2	25.3	23.3, 27.4
Tumor behavior				
Benign/borderline	2.7	2.4, 3.0	15.4	14.3, 16.6
Malignant	1.6	1.4, 1.9	7.9	7.1, 8.8
Unspecified	6.1	5.7, 6.6	–	
Tumor behavior by sex				
Men benign/borderline	2.0	1.7, 2.4	11.0	9.7, 12.5
Men malignant	1.9	1.6, 2.3	10.1	8.8, 11.5
Men unspecified	5.3	4.8, 5.9	–	–
Women benign/border	3.2	2.8, 3.6	19.1	17.4, 21.0
Women malignant	1.4	1.2, 1.7	6.1	5.1, 7.2
Women unspecified	5.9	5.3, 6.5	–	

*AIR* age-adjusted incidence rate per 100,000 person-years (adjusted to the US 2000 standard population), *95% CI* 95% confidence intervals (based on the gamma distribution proposed by Tiwari et al. [17])

### Incidence rates and distribution of histology subtypes

**Fig. 1:**
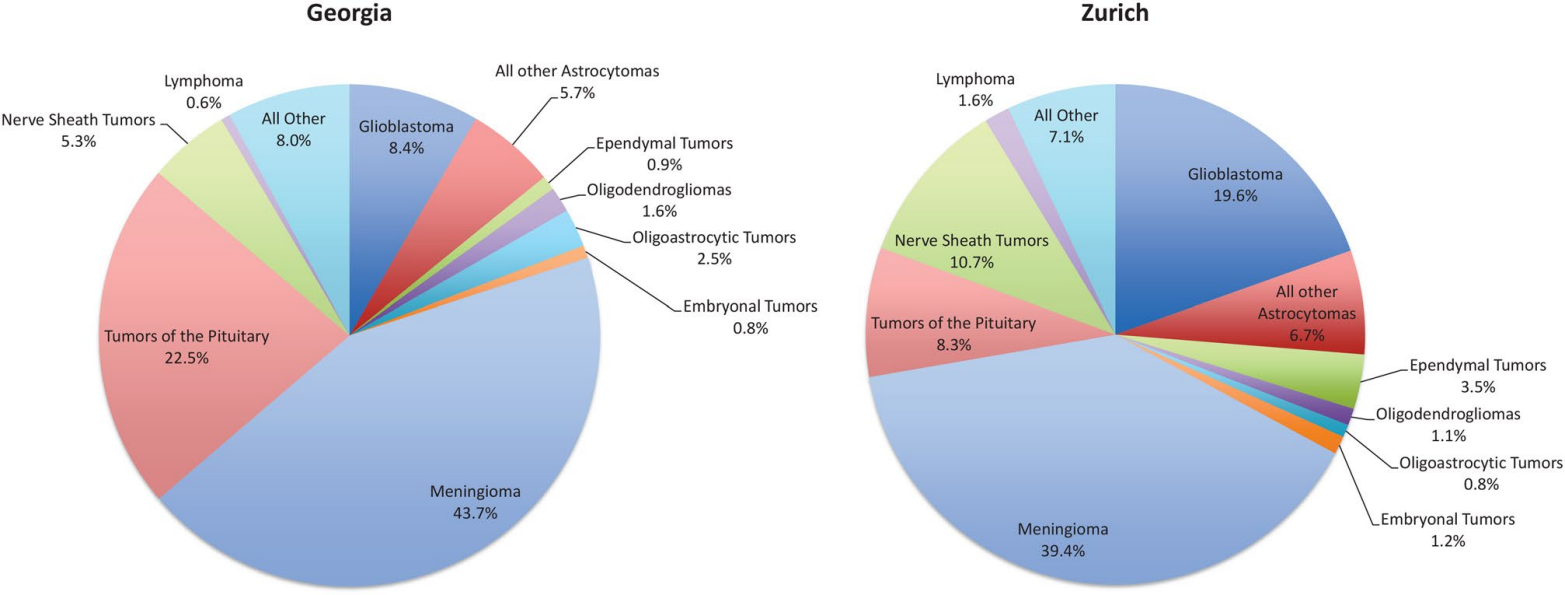
Proportion of different histology subtypes for Georgia and Zurich (Switzerland), March 1, 2009 to February 29, 2012

Crude and age-adjusted incidence rates for each histology group according to WHO 2007 histological classification are shown in Table 3 for Georgia and Table 4 for Zurich. The most frequent histology type were meningiomas in both regions (39.4% in Zurich versus 43.7% in Georgia, Fig. 1), followed by glioblastomas in Zurich (19.6%)/pituitary tumors in Georgia (22.5%), and nerve sheath tumors in Zurich (10.7%)/glioblastoma in Georgia (8.4%).

**Fig. 2:**
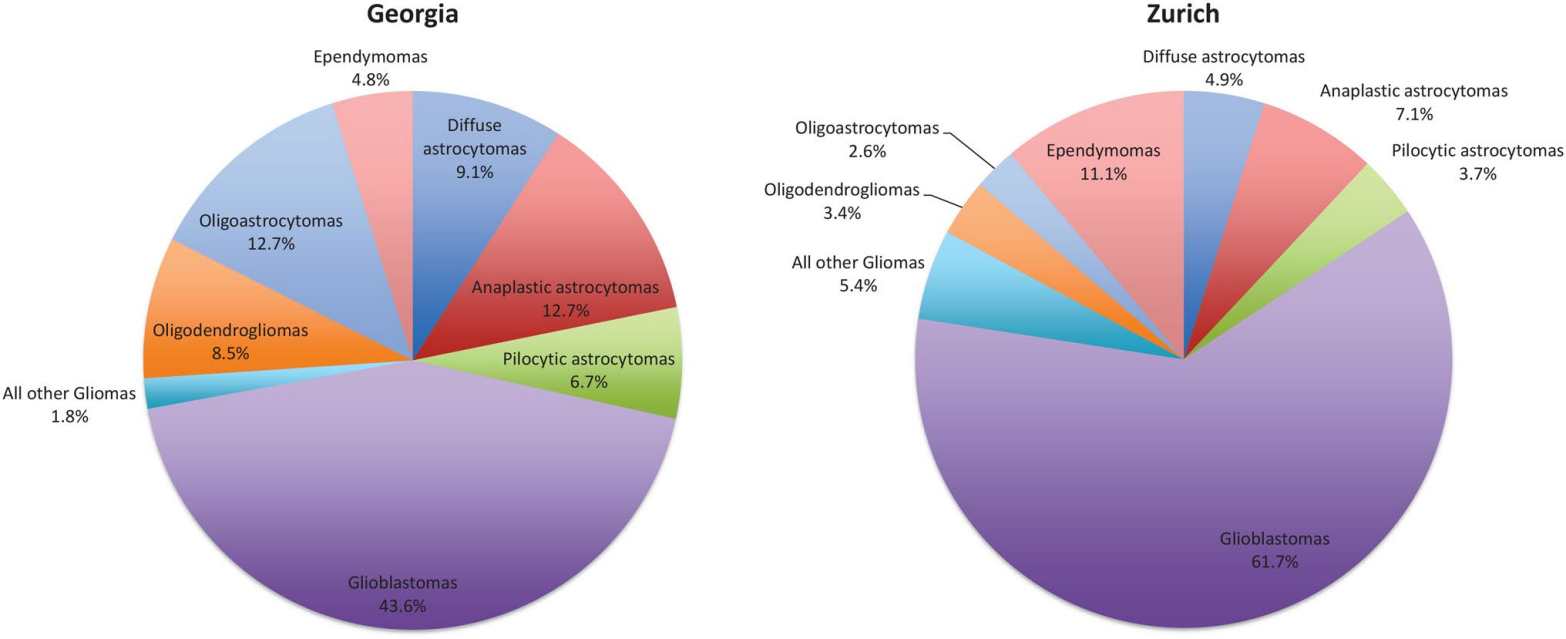
Proportion of different glioma subtypes for Georgia and Zurich (Switzerland), March 1, 2009 to February 29, 2012

Among gliomas, glioblastoma was the most frequent type (61.7% in Zurich versus 43.6% in Georgia, respectively; Fig. 2). Glioblastoma combined with all other astrocytoma types accounted for approximately three-fourth of gliomas in both regions (77.4% in Zurich versus 72.1% in Georgia).

### Incidence rates by sex and histology subtypes

Overall incidence of brain tumors was higher in women than in men, although this difference was only significant in Zurich (25.3 vs 21.1 per 100,000 person-years; Tables 3 (Georgia) and 4 (Zurich)). The incidence rates for all neuroepithelial tumors were higher in men than in women; however, the difference was only statistically significant for Zurich. A higher incidence rate in men was also observed for glioblastomas, but the difference was not statistically significant in either region. In both regions, the incidence rates for meningiomas were higher in women than in men, but only statistically significant in Zurich. We found no significant difference between men and women for pituitary adenomas.

**Table 3:** Crude and age-adjusted incidence rates of brain/CNS tumors by sex, according to histology group, for Georgia, March 1, 2009 to February 29, 2012

Group	Specific histology	WHO histology code	N	%	CIR	AIR (95% CI)	Females				Males			
							N	%	CIR	AIR (95% CI)	N	%	CIR	AIR (95% CI)
Neuroepithelial tumors			180	12.2	1.35	1.30 (1.12, 1.51)	94	11.7	1.35	1.24 (0.99, 1.52)	84	14.3	1.33	1.34 (1.07, 1.67)
	Pilocytic astrocytoma	9421	11	0.7	0.08	0.10 (0.05, 0.17)	5	0.6	0.07	0.08 (0.03, 0.18)	6	1.0	0.09	0.11 (0.04, 0.25)
	Diffuse astrocytoma	9400, 9411, 9420	15	1.0	0.11	0.10 (0.06, 0.17)	7	0.9	0.10	0.10 (0.04, 0.20)	8	1.4	0.13	0.12 (0.05, 0.24)
	Anaplastic astrocytoma	9401	21	1.4	0.16	0.15 (0.09, 0.23)	11	1.4	0.16	0.15 (0.07, 0.27)	10	1.7	0.16	0.15 (0.07, 0.28)
	Glioblastoma	9440, 9441, 9442/3	72	4.9	0.54	0.50 (0.39, 0.63)	35	4.4	0.50	0.42 (0.29, 0.59)	37	6.3	0.59	0.59 (0.42, 0.82)
	Other astrocytomas	9380, 9381, 9384, 9424, 9425	2	0.1	0.02	0.01 (0.00, 0.05)	1	0.1	0.01	0.01 (0.00, 0.08)	1	0.2	0.02	0.02 (0.00, 0.10)
	Oligodendroglioma	9450	9	0.6	0.07	0.06 (0.03, 0.12)	5	0.6	0.07	0.06 (0.02, 0.14)	4	0.7	0.06	0.06 (0.02, 0.17)
	Anaplastic oligodendroglioma	9451	5	0.3	0.04	0.04 (0.01, 0.09)	2	0.2	0.03	0.03 (0.00, 0.11)	3	0.5	0.05	0.05 (0.01, 0.14)
	Oligoastrocytoma	9382	21	1.4	0.16	0.15 (0.09, 0.24)	13	1.6	0.19	0.18 (0.09, 0.31)	8	1.4	0.13	0.12 (0.05, 0.25)
	Ependymoma	9383, 9391, 9392, 9394	8	0.5	0.06	0.06 (0.02, 0.12)	7	0.9	0.10	0.09 (0.04, 0.19)	1	0.2	0.02	0.02 (0.00, 0.11)
	Neuronal—glial	8680, 9390, 9413, 9492, 9493, 9505, 9506	9	0.6	0.07	0.06 (0.03, 0.12)	6	0.7	0.09	0.08 (0.03, 0.18)	1	0.2	0.02	0.01 (0.00, 0.09)
	Embryonal—medulloblastoma	9470, 9471, 9473, 9508	7	0.5	0.05	0.06 (0.03, 0.13)	2	0.2	0.03	0.03 (0.00, 0.12)	5	0.9	0.08	0.09 (0.03, 0.21)
Tumors of cranial and spinal nerves	Neurinoma	9560	45	3.0	0.34	0.33 (0.24, 0.44)	30	3.7	0.43	0.39 (0.26, 0.56)	14	2.4	0.22	0.24 (0.13, 0.40)
Tumors of meninges			410	27.8	3.05	2.81 (2.54, 3.10)	262	32.6	3.70	3.23 (2.84, 3.66)	125	21.3	1.96	1.92 (1.60, 2.30)
	Meningioma	9530, 9531, 9532, 9533, 9534, 9537, 9538, 9539	374	25.3	2.78	2.56 (2.30, 2.84)	243	30.3	3.43	2.97 (2.60, 3.39)	110	18.7	1.73	1.70 (1.39, 2.05)
	Mesenchymal—lipoma, haemangioma	8815, 8850, 8861, 9120, 9133, 9150, 9180, 9220, 9364	25	1.7	0.19	0.18 (0.11, 0.26)	14	1.7	0.20	0.19 (0.10, 0.32)	10	1.7	0.16	0.15 (0.07, 0.28)
	Haemangioblastoma	9161	11	0.7	0.08	0.08 (0.04, 0.14)	5	0.6	0.07	0.07 (0.02, 0.16)	5	0.9	0.08	0.08 (0.02, 0.19)
Lymphomas	Lymphoma	9590, 9680	5	0.3	0.04	0.04 (0.01, 0.09)	3	0.4	0.04	0.04 (0.01, 0.12)	2	0.3	0.03	0.03 (0.00, 0.13)
Germ cell	Germinoma	9064, 9080, 9084	4	0.3	0.03	0.03 (0.01, 0.07)	1	0.1	0.01	0.01 (0.00, 0.08)	3	0.5	0.05	0.05 (0.01, 0.15)
Tumors of the sellar region			211	14.3	1.58	1.52 (1.32, 1.75)	125	15.6	1.79	1.73 (1.44, 2.06)	69	11.7	1.08	1.05 (0.82, 1.34)
	Pituitary adenoma	8271, 8272	192	13.0	1.44	1.38 (1.19, 1.59)	115	14.3	1.65	1.57 (1.30, 1.89)	61	10.4	0.95	0.93 (0.71, 1.21)
	Craniopharyngioma	9350	19	1.3	0.14	0.14 (0.09, 0.22)	10	1.2	0.14	0.15 (0.07, 0.28)	8	1.4	0.13	0.12 (0.05, 0.24)
Unclassified	Unclassified	8000	621	42.3	4.66	4.45 (4.10, 4.82)	288	35.9	4.12	3.83 (3.39, 4.31)	291	49.5	4.59	4.56 (4.05, 5.13)
Total			1476	100	11.05	10.48 (9.94, 11.04)	803	100	11.44	10.47 (9.75, 11.24)	588	100	9.26	9.20 (8.47, 9.99)

Age unknown: *N* = 8

Sex unknown: *N* = 85

*AIR* age-adjusted incidence rate per 100,000 person-years (adjusted to the US 2000 standard population), *95% CI* 95% confidence intervals (based on the gamma distribution proposed by Tiwari et al. [17]), *CIR* crude incidence rate

**Table 4:** Crude and age-adjusted incidence rates of brain/CNS tumors by sex, according to histology group, for Zurich (Switzerland), March 1, 2009 to February 29, 2012

Group	Specific histology	WHO histology code	N	%	CIR	AIR (95% CI)	Females				Males			
							N	%	CIR	AIR (95% CI)	N	%	CIR	AIR (95% CI)
Neuroepithelial			377	34.1	9.23	8.37 (7.52, 9.29)	148	23.3	7.16	6.49 (5.45, 7.68)	229	48.7	11.35	10.57 (9.22, 12.07)
	Pilocytic astrocytoma	9421	13	1.2	0.32	0.43 (0.23, 0.73)	4	0.6	0.19	0.27 (0.07, 0.66)	9	1.9	0.45	0.59 (0.27, 1.10)
	Diffuse astrocytoma	9400, 9411, 9420	17	1.5	0.42	0.37 (0.21, 0.60)	7	1.1	0.34	0.34 (0.13, 0.71)	10	2.1	0.50	0.42 (0.20, 0.79)
	Anaplastic astrocytoma	9401	25	2.3	0.61	0.58 (0.37, 0.86)	10	1.6	0.48	0.47 (0.22, 0.87)	15	3.2	0.74	0.70 (0.39, 1.17)
	Glioblastoma	9440, 9441, 9442/3	216	19.6	5.29	4.32 (3.76, 4.95)	83	13.1	4.02	3.20 (2.53, 3.99)	133	28.3	6.59	5.69 (4.76, 6.77)
	Other astrocytomas	9380, 9381, 9384, 9424, 9425	19	1.7	0.47	0.44 (0.26, 0.70)	10	1.6	0.48	0.50 (0.22, 0.94)	9	1.9	0.45	0.42 (0.19, 0.81)
	Oligodendroglioma	9450	5	0.5	0.12	0.11 (0.03, 0.26)	2	0.3	0.10	0.09 (0.01, 0.34)	3	0.6	0.15	0.12 (0.02, 0.39)
	Anaplastic oligodendroglioma	9451	7	0.6	0.17	0.15 (0.06, 0.31)	3	0.5	0.15	0.13 (0.03, 0.40)	4	0.9	0.20	0.17 (0.05, 0.46)
	Oligoastrocytoma	9382	9	0.8	0.22	0.20 (0.09, 0.38)	4	0.6	0.19	0.17 (0.05, 0.45)	5	1.1	0.25	0.22 (0.07, 0.53)
	Ependymoma	9383, 9391, 9392, 9394	39	3.5	0.95	0.96 (0.68, 1.32)	12	1.9	0.58	0.63 (0.32, 1.11)	27	5.7	1.34	1.31 (0.86, 1.92)
	Neuronal—glial	8680, 9390, 9413, 9492, 9493, 9505, 9506	14	1.3	0.34	0.40 (0.21, 0.67)	7	1.1	0.34	0.32 (0.13, 0.68)	7	1.5	0.35	0.46 (0.18, 0.92)
	Embryonal—medulloblastoma	9470, 9471, 9473, 9508	13	1.2	0.32	0.42 (0.22, 0.72)	6	0.9	0.29	0.38 (0.13, 0.81)	7	1.5	0.35	0.46 (0.19, 0.93)
Tumours of cranial and spinal nerves	Neurinoma	9560	118	10.7	2.89	2.50 (2.06, 3.00)	61	9.6	2.95	2.51 (1.91, 3.24)	57	12.1	2.82	2.45 (1.85, 3.19)
Tumours of meninges			449	40.7	10.99	9.04 (8.22, 9.93)	346	54.6	16.75	13.02 (11.66, 14.51)	103	21.9	5.10	4.45 (3.63, 5.42)
	Meningioma	9530, 9531, 9532, 9533, 9534, 9537, 9538, 9539	435	39.4	10.65	8.71 (7.90, 9.58)	340	53.6	16.46	12.72 (11.38, 14.19)	95	20.2	4.71	4.06 (3.28, 4.98)
	Mesenchymal—lipoma, haemangioma	8815, 8850, 8861, 9120, 9133, 9150, 9180, 9220, 9364	3	0.3	0.07	0.07 (0.01, 0.21)	2	0.3	0.10	0.10 (0.01, 0.36)	1	0.2	0.05	0.05 (0.00, 0.27)
	Haemangioblastoma	9161	11	1.0	0.27	0.26 (0.13, 0.48)	4	0.6	0.19	0.20 (0.05, 0.52)	7	1.5	0.35	0.34 (0.14, 0.72)
Lymphomas	Lymphoma	9590, 9680	18	1.6	0.44	0.37 (0.22, 0.59)	7	1.1	0.34	0.31 (0.12, 0.65)	11	2.3	0.55	0.49 (0.24, 0.89)
Germ cell	Germinoma	9064, 9080, 9084,	5	0.5	0.12	0.13 (0.04, 0.31)	1	0.2	0.05	0.04 (0.00, 0.26)	4	0.9	0.20	0.22 (0.06, 0.55)
Tumours of the sellar region			92	8.3	2.25	1.92 (1.54, 2.36)	44	6.9	2.13	1.80 (1.30, 2.44)	48	10.2	2.38	2.11 (1.55, 2.81)
	Pituitary adenoma	8271, 8272	92	8.3	2.25	1.92 (1.54, 2.36)	44	6.9	2.13	1.80 (1.30, 2.44)	48	10.2	2.38	2.11 (1.55, 2.81)
	Craniopharyngioma	9350	0	0	0	0	0	0	0	0	0	0	0	0
Unclassified	Unclassified	8000	45	4.1	1.10	0.96 (0.69, 1.30)	27	4.3	1.31	1.10 (0.70, 1.64)	18	3.8	0.89	0.83 (0.49, 1.33)
Total			1104	100	27.03	23.29 (21.91, 24.73)	634	100	30.69	25.27 (23.29, 27.39)	470	100	23.29	21.12 (19.22, 23.16)

*AIR* age-adjusted incidence rate per 100,000 person-years (adjusted to the US 2000 standard population), *95% CI* 95% confidence intervals (based on the gamma distribution proposed by Tiwari et al. [17]), *CIR* crude incidence rate

### Age distribution

In Zurich, incidence rates by age were clearly increasing for all tumors and for meningiomas specifically, which was not the case in Georgia (Online Resource 2). Nevertheless, we observed a plateau of rates between the age of 40 and 80 years except for meningiomas and glioblastomas.

## Discussion

Our study showed large differences in the incidence of malignant and benign brain/CNS tumors between Georgia, a lower middle-income country, and Zurich (Switzerland), situated in a high-income country. Differences in incidence rates with higher rates in higher income countries than in low- and middle-income countries have been reported previously [3, 5, 19]. According to a systematic analysis of the global burden of brain/CNS cancer, the percentage change in age-standardized rates between 1990 and 2016 was clearly higher in high-income compared to low-income countries [20].

These differences may be attributable to population genetics, oncogenic environmental exposures, healthcare access, diagnostic practice, or registry reporting [5]. Specifically, differences in case ascertainment likely explain a large part of the reported variation, with patients in lower-income countries having reduced access to advanced imaging technology [4, 5]. Switzerland has a mandatory universal basic health insurance and high medical and technological standards. In Georgia, after 2006 governmental budget was used to finance health care for the poorest households only. A medical insurance program was rolled out nation-wide in 2008 also targeting households registered as living below the poverty line. For those above the poverty line, state-funded programs were introduced offering specific services, which partially include brain tumor (surgery and palliative care). The government introduced a universal health care program extending the population and services covered only in 2013. Thus, the healthcare system in Georgia was not universal during the study period (2009–2012), which may have biased data collection due to the limited resources.

The most frequent histology type among all registered brain/CNS tumors in both regions were meningiomas (43.7% in Georgia and 39.4% in Zurich). However, the second frequent histology subtype in Georgia were tumors of the pituitary (22.5% versus 8.3% in Zurich), while in Zurich glioblastomas ranked second (19.6% versus 8.4% in Georgia). The lower rate of verified glioblastoma in Georgia may be due to a lack of services for the older population, specifically those living in rural areas, including a lack of information, caregiving, diagnostic services, and generally limited resources. The proportional distribution of glioma subtypes also differed between the regions, with higher proportions of glioblastomas and ependymomas in Zurich und higher proportions of astrocytomas and oligodendrogliomas in Georgia. We can speculate that histologically unclassified tumors in Georgia may include cases of glioblastoma, which usually affect people in the highest age group. On the other hand, astrocytomas and oligodendrogliomas are most frequently observed in individuals aged between 30 and 40 years, where more resources, support and attention are available than for older population groups.

The comparison of the incidence rates by histological group (Tables 3 and 4) may be influenced by the high proportion of unclassified tumors in Georgia (621 out of 1476). However, excluding unclassified tumors would considerably decrease the overall incidence rate in Georgia and would not be in accordance with the main aim of this study to compare overall incidence of brain/CNS tumors between Georgia and Switzerland.

While the age-stratified incidence rates increased with age in Zurich with highest rates in the oldest age group (≥ 80 years), we observed the highest incidence rates in Georgia for individuals aged 60–79 years. This may also be related to the lack of service for the older population, as mentioned above, which results in an underestimation of cases in that age group. In addition, social stigma, geopolitical issues, and cultural norms may limit access to adequate cancer care in Georgia, including cancer detection, diagnosis and treatment, especially in the elderly. Therefore, cultural differences, in addition to cancer care infrastructure and workforce capacity constraints, need to be taken into account when interpreting the differences between the two regions.

The ratio of benign to malignant brain/CNS tumors was 1.946 for Zurich, which is similar to the ratio of 2.114 reported for the US [5]. For Georgia, this ratio was lower (1.656), however, there were quite a few tumors with unspecified behavior which may have influenced this ratio.

### Comparison with other studies

The comparison of incidence rates between different countries and regions using other published studies is not straightforward due to different standard populations used for age-standardization and differences regarding the definition and inclusion of brain/CNS tumors.

The incidence rate of malignant brain/CNS tumors in Zurich (7.9 [95% CI 7.1–8.8] per 100,000 person-years using the US standard population) is comparable to rates reported for Europe (6.76 [95% CI 6.71–6.80]) [5], and the one adjusted to the WHO standard population (6.9 [95% CI 6.1–7.7]) is comparable to rates reported for Western Europe (5.84 [95% CI 5.77–5.92]) [19]. On the other hand, the WHO age-standardized incidence rate for Georgia (malignant brain/CNS tumors only, 1.5 [95% CI 1.3–1.7]) is considerably lower compared to rates reported for Eastern Europe and Central Asia (4.82 [95% CI 4.76–4.89]) [19], but only slightly lower than reported for Iran (2.74 [95% CI 2.62–2.86]) [21].

The overall age-standardized incidence rate for all brain/CNS tumors in Zurich (23.3 [95% CI 21.9–24.7] per 100,000 person-years) is in a similar range as reported in the CBTRUS report for the United States (21.42 [95% CI 21.35–21.49]) [12] but higher than in France (15.95 per 100,000 person-years) [22]. However, some tumors were excluded from the French data (such as pituitary tumors) and the time period investigated in France was earlier (2000–2007) [22].

The overall age-standardized incidence rate for all brain/CNS tumors in Georgia (10.5 [95% CI 9.9–11.0] per 100,000 person-years) is comparable to the mean worldwide rate of 10.82 (95% CI 8.63–13.56) [23]. In that study, results varied between 1.88 per 100,000 person-years for Jordan and 25.95 per 100,000 person-years for Italy [23]. Thus, while the rate for Georgia is in line with the world average, the rate for Zurich is in the range of countries with the highest reported rates.

Using the world standard population for adjustment, the age-standardized incidence rate for malignant brain/CNS tumors of 6.5 (95% CI 5.8–7.3; Online Resource 1) for Zurich is comparable to rates reported for other parts of Switzerland (7.7 [95% CI 6.9–8.4] for men and 5.5 [95% CI 4.8–6.1] for women) and other parts of Western Europe [3]. On the other hand, the age-standardized incidence rate for Georgia (using the world standard population) of 1.4 (95% CI 1.2–1.6) was again considerably lower than rates reported for other Eastern European countries (including Russia; between 6.2 and 9.8 for men and between 4.5 and 9.2 for women) [3] and Estonia (8.5 [95% CI 8.0–8.9]) [24].

Using the European standard population for adjustment, the rates for Zurich (14.7 [95% CI 13.7–15.9] for benign and 7.9 [95% CI 7.1–8.8] for malignant tumors) were slightly higher compared to data from a larger part of Switzerland (9.69 for benign, 6.64 for malignant) [4]. The difference in incidence rates for benign/borderline versus malignant tumors in men and women has also been reported for other parts of Switzerland [4].

### Strengths and limitations

This study allows for the direct comparison between brain/CNS tumor rates in a lower middle-income country and a high-income country using the same definitions and criteria.

A limitation is the relatively low proportion of histologically verified cases in Georgia (37%), which is considerably lower than in Zurich (64%) and other countries such as France (79%) [22], and the US according to CBTRUS (62% overall, 85% for malignant, 50% for non-malignant tumors) [12]. However, lower rates of histologically verified cases have been reported in other European countries such as Croatia (57.2%) [25], Italy (49%) [26] or Spain (59%) [27]. We can assume that primary brain tumor cases found in relevant medical documentations are almost complete in the Georgian registry, but the number of histologically unclassified cases is high. These were mostly registered from radiological (CT, MRI) reports, but were not available in any other hospital data. In a few cases, the histology reports were just missing in medical records at the moment of data retrieving. It is therefore likely that the true incidence is higher than the reported rates in Georgia, primarily in subgroups (by behavior or histology). Finally, tumors of topography C75.2 were included for Georgia but are not systematically registered in Zurich and were thus excluded for Zurich. However, these only accounted for 1.3% of all brain/CNS tumors in Georgia, and because we wanted to be as complete as possible for the Georgian data, we included them.

## Conclusions

We observed considerably higher incidence rates of brain/CNS tumors in Zurich compared to Georgia, both for benign and malignant tumors. These observations are in line with other publications reporting higher rates in high-income countries and lower rates in low- and middle-income countries [3, 5, 19]. Meningiomas accounted for the largest proportion in both regions (around 40%), whereas the second most frequent tumors were pituitary tumors in Georgia, but glioblastomas in Zurich (around 20% each). The frequency distribution may be related to differences in diagnosing techniques and the population age structure. The incidence rates by age group increased in Zurich with highest rates in the oldest age group (≥ 80 years), but the highest rates in Georgia were seen for individuals aged 60–79 years, which may reflect the lack of service for the older rural population in Georgia, resulting in an underestimation of cases in the oldest age group.

## Electronic supplementary material

Below is the link to the electronic supplementary material.

Supplementary file 1 (PDF 117 kb)

Supplementary file 2 (PDF 39 kb)

## Data Availability

The data are not publicly available.

## Code availability

The STATA code is available upon request from the corresponding author.

## References

[1] Bondy ML, Scheurer ME, Malmer B, Barnholtz-Sloan JS, Davis FG, Il'yasova D, Kruchko C, McCarthy BJ, Rajaraman P, Schwartzbaum JA, Sadetzki S, Schlehofer B, Tihan T, Wiemels JL, Wrensch M, Buffler PA, Brain Tumor Epidemiology C (2008). Brain tumor epidemiology: consensus from the Brain Tumor Epidemiology Consortium. Cancer.

[2] Arndt V, Feller A, Hauri D, Heusser R, Junker C, Kuehni C, Lorez M, Pfeiffer V, Roy E, Schindler M (2016). Swiss Cancer Report 2015: current situation and developments.

[3] Miranda-Filho A, Pineros M, Soerjomataram I, Deltour I, Bray F (2017). Cancers of the brain and CNS: global patterns and trends in incidence. Neuro Oncol.

[4] Lorez M, Nanieva R, Arndt V, Rohrmann S, Group NW (2018). Benign and malignant primary brain tumours in the Swiss population (2010–2014). Swiss Cancer Bull.

[5] Bell JS, Koffie RM, Rattani A, Dewan MC, Baticulon RE, Qureshi MM, Wahjoepramono EJ, Rosseau G, Park K, Nahed BV (2019). Global incidence of brain and spinal tumors by geographic region and income level based on cancer registry data. J Clin Neurosci.

[6] Statistical Office of the Canton of Zurich (2019). Statistisches Jahrbuch des Kantons Zürich 2019 (Statistical Annual Report of the Canton of Zurich 2019).

[7] Wanner M, Matthes KL, Korol D, Dehler S, Rohrmann S (2018). Indicators of data quality at the Cancer Registry Zurich and Zug in Switzerland. Biomed Res Int.

[8] Lorez M, Bordoni A, Bouchardy C, Bulliard JL, Camey B, Dehler S, Frick H, Konzelmann I, Maspoli M, Mousavi SM, Rohrmann S, Arndt V (2017). Evaluation of completeness of case ascertainment in Swiss cancer registration. Eur J Cancer Prev.

[9] Gigineishvili D, Shengelia N, Shalashvili G, Rohrmann S, Tsiskaridze A, Shakarishvili R (2013). Primary brain tumour epidemiology in Georgia: first-year results of a population-based study. J Neurooncol.

[10] Gigineishvili D, Gigineishvili T, Tsiskaridze A, Shakarishvili R (2014). Incidence rates of the primary brain tumours in Georgia: a population-based study. BMC Neurol.

[11] Louis DN, Ohgaki H, Wiestler OD, Cavenee WK, Burger PC, Jouvet A, Scheithauer BW, Kleihues P (2007). The 2007 WHO classification of tumours of the central nervous system. Acta Neuropathol.

[12] Ostrom QT, Gittleman H, Liao P, Rouse C, Chen Y, Dowling J, Wolinsky Y, Kruchko C, Barnholtz-Sloan J (2014). CBTRUS statistical report: primary brain and central nervous system tumors diagnosed in the United States in 2007–2011. Neuro Oncol.

[13] Klein RJ, Schoenborn CA (2001). Age adjustment using the 2000 projected US population. Healthy People 2010 Stat Notes.

[14] Ahmad OB, Boschi-Pinto C, Lopez AD, Murray CJL, Lozano R, Inoue M (2001) Age standardization of rates: a new WHO standard. GPE Discussion Paper Series, vol 31. WHO

[15] Waterhouse JAH, Muir CS, Correa P, Powell J (1976) Cancer incidence in five continents, vol. III IARC Scientific Publications No. 15. IARC, Lyon

[16] Consonni D, Coviello E, Buzzoni C, Mensi C (2012). A command to calculate age-standardized rates with efficient interval estimation. Stata J.

[17] Tiwari RC, Clegg LX, Zou Z (2006). Efficient interval estimation for age-adjusted cancer rates. Stat Methods Med Res.

[18] Boyle P, Parkin DM (1991). Cancer registration: principles and methods. Statistical methods for registries. IARC Sci Publ.

[19] Leece R, Xu J, Ostrom QT, Chen Y, Kruchko C, Barnholtz-Sloan JS (2017). Global incidence of malignant brain and other central nervous system tumors by histology, 2003–2007. Neuro Oncol.

[20] Patel AP, Fisher JL, Nichols E, Abd-Allah F, Abdela J, Abdelalim A, Abraha HN, Agius D, Alahdab F (2019). Global, regional, and national burden of brain and other CNS cancer, 1990–2016: a systematic analysis for the Global Burden of Disease Study 2016. Lancet Neurol.

[21] Jazayeri SB, Rahimi-Movaghar V, Shokraneh F, Saadat S, Ramezani R (2013). Epidemiology of primary CNS tumors in Iran: a systematic review. Asian Pac J Cancer Prev.

[22] Baldi I, Gruber A, Alioum A, Berteaud E, Lebailly P, Huchet A, Tourdias T, Kantor G, Maire JP, Vital A, Loiseau H, Gironde TRG (2011). Descriptive epidemiology of CNS tumors in France: results from the Gironde Registry for the period 2000–2007. Neuro Oncol.

[23] de Robles P, Fiest KM, Frolkis AD, Pringsheim T, Atta C, St Germaine-Smith C, Day L, Lam D, Jette N (2015). The worldwide incidence and prevalence of primary brain tumors: a systematic review and meta-analysis. Neuro Oncol.

[24] Liigant A, Asser T, Kulla A, Kaasik AE (2000). Epidemiology of primary central nervous system tumors in Estonia. Neuroepidemiology.

[25] Georgakis MK, Panagopoulou P, Papathoma P, Tragiannidis A, Ryzhov A, Zivkovic-Perisic S, Eser S, Taraszkiewicz L, Sekerija M, Zagar T, Antunes L, Zborovskaya A, Bastos J, Florea M, Coza D, Demetriou A, Agius D, Strahinja RM, Sfakianos G, Nikas I, Kosmidis S, Razis E, Pourtsidis A, Kantzanou M, Dessypris N, Petridou ET (2017). Central nervous system tumours among adolescents and young adults (15–39 years) in Southern and Eastern Europe: registration improvements reveal higher incidence rates compared to the US. Eur J Cancer.

[26] Caldarella A, Crocetti E, Paci E (2011). Is the incidence of brain tumors really increasing? A population-based analysis from a cancer registry. J Neurooncol.

[27] Fuentes-Raspall R, Vilardell L, Perez-Bueno F, Joly C, Garcia-Gil M, Garcia-Velasco A, Marcos-Gragera R (2011). Population-based incidence and survival of central nervous system (CNS) malignancies in Girona (Spain) 1994–2005. J Neurooncol.

