# Supplementary material for: Geographical variation in malignant and benign/borderline brain and CNS tumor incidence: a comparison between a high-income and a middle-income country
Source: J Neurooncol. 2020 Aug 19;149(2):273–82. doi: 10.1007/s11060-020-03595-5 (PMC7541360; doi:10.1007/s11060-020-03595-5)
Supplement: Supplementary file 1 — (PDF 117 kb) [file 11060_2020_3595_MOESM1_ESM.pdf]

Geographical variation in malignant and benign/borderline brain and CNS tumor incidence: A comparison between a high-income and a middle-income country

Journal of Neuro-Oncology

Miriam Wanner, Sabine Rohrmann, Dimitri Korol, Nino Shenglia, Teimuraz Gigineishvili, David Gigineishvili

Corresponding author:
Miriam Wanner: miriam.wanner@uzh.ch

Online Resource 1 (Table). Age-adjusted incidence rates of brain and cns tumors in Georgia and Zurich (Switzerland), March 1, 2009 to February 29, 2012, using different standard populations

	World standard population				WHO standard population				European standard population (1976)			
	Georgia		Zurich		Georgia		Zurich		Georgia		Zurich	
	AIR	95% CI	AIR	95% CI	AIR	95% CI	AIR	95% CI	AIR	95% CI	AIR	95% CI
Overall	9.1	8.6, 9.6	18.1	16.9, 19.4	9.5	9.0, 10.0	19.5	18.2, 20.7	10.9	10.3, 11.5	22.7	21.3, 24.1
Sex												
Men	8.0	7.3, 8.7	16.7	15.1, 18.5	8.3	7.6, 9.0	17.8	16.2, 19.7	9.5	8.8, 10.4	20.6	18.8, 22.6
Women	9.1	8.4, 9.8	19.4	17.7, 21.3	9.5	8.8, 10.2	21.0	19.2, 22.9	10.9	10.2, 11.7	24.6	22.6, 26.7
Tumor behavior												
Benign/borderline	2.4	2.1, 2.6	11.6	10.6, 12.6	2.5	2.2, 2.8	12.6	11.6, 13.6	2.8	2.5, 3.1	14.7	13.7, 15.9
Malignant	1.4	1.2, 1.6	6.5	5.8, 7.3	1.5	1.3, 1.7	6.9	6.1, 7.7	1.7	1.5, 1.9	7.9	7.1, 8.8
unspecified	5.3	4.9, 5.7	-		5.5	5.1, 5.9	-		6.4	5.9, 6.8	-	
Tumor behavior and sex												
Men benign/borderline	1.7	1.4, 2.1	8.8	7.6, 10.2	1.8	1.5, 2.2	9.4	8.2, 10.8	2.0	1.7, 2.4	10.7	9.4, 12.2
Men malignant	1.6	1.3, 1.9	7.9	6.8, 9.1	1.7	1.4, 2.0	8.4	7.3, 9.7	1.9	1.6, 2.3	9.9	8.6, 11.3
Men unspecified	4.6	4.1, 5.2	-		4.8	4.3, 5.4	-		5.6	5.0, 6.2	-	
Women benign/borderl.	2.7	2.4, 3.1	14.1	12.7, 15.6	2.9	2.5, 3.3	15.5	14.0, 17.1	3.3	2.9, 3.8	18.4	16.7, 20.2
Women malignant	1.2	1.0, 1.5	5.3	4.3, 6.4	1.3	1.1, 1.6	5.5	4.5, 6.6	1.5	1.2, 1.8	6.2	5.2, 7.3
Women unspecified	5.1	4.6, 5.6	-		5.3	4.8, 5.9	-		6.1	5.5, 6.7	-	

Abbreviations: AIR, age-adjusted incidence rate per 100,000 person-years (adjusted to different standard populations); 95% CI, 95% confidence intervals (based on the gamma distribution proposed by Tiwari et al. 2006 [17]
