# Supplementary material for: Geographical variation in malignant and benign/borderline brain and CNS tumor incidence: a comparison between a high-income and a middle-income country
Source: J Neurooncol. 2020 Aug 19;149(2):273–82. doi: 10.1007/s11060-020-03595-5 (PMC7541360; doi:10.1007/s11060-020-03595-5)
Supplement: Supplementary file 2 — (PDF 39 kb) [file 11060_2020_3595_MOESM2_ESM.pdf]

Geographical variation in malignant and benign/borderline brain and CNS tumor incidence: A comparison between a high-income and a middle-income country

Journal of Neuro-Oncology

Miriam Wanner, Sabine Rohrmann, Dimitri Korol, Nino Shenglia, Teimuraz Gigineishvili, David Gigineishvili

Corresponding author:

Miriam Wanner: miriam.wanner@uzh.ch

Online Resource 2 (Figure). Age-standardized incidence rates for selected histology subtypes by age group for Georgia and Zurich (Switzerland), March 1, 2009 to February 29, 2012

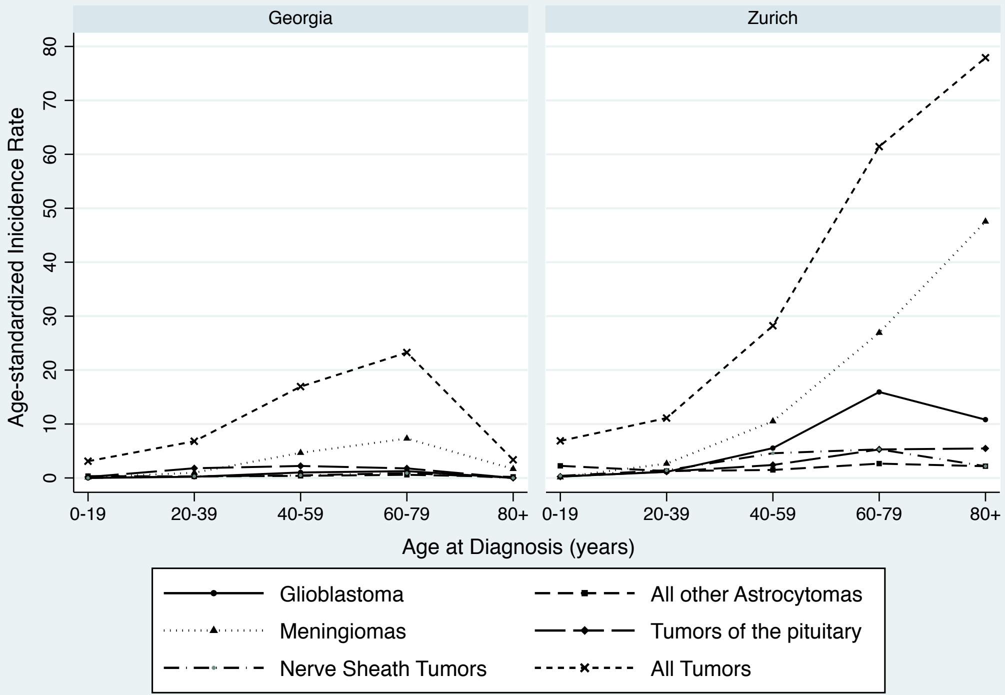

Georgia
Zurich
Age-standardized Inicidence Rate
0
10
20
30
40
50
60
70
80
0-19
20-39
40-59
60-79
80+
Age at Diagnosis (years)
Glioblastoma
All other Astrocytomas
Meningiomas
Tumors of the pituitary
Nerve Sheath Tumors
All Tumors
